# Supplementary figures and images for: Transmission competence of a new mesonivirus, Yichang virus, in mosquitoes and its interference with representative flaviviruses
Source: PLoS Negl Trop Dis. 2020 Nov 30;14(11):e0008920. doi: 10.1371/journal.pntd.0008920 (PMC7738168; doi:10.1371/journal.pntd.0008920)

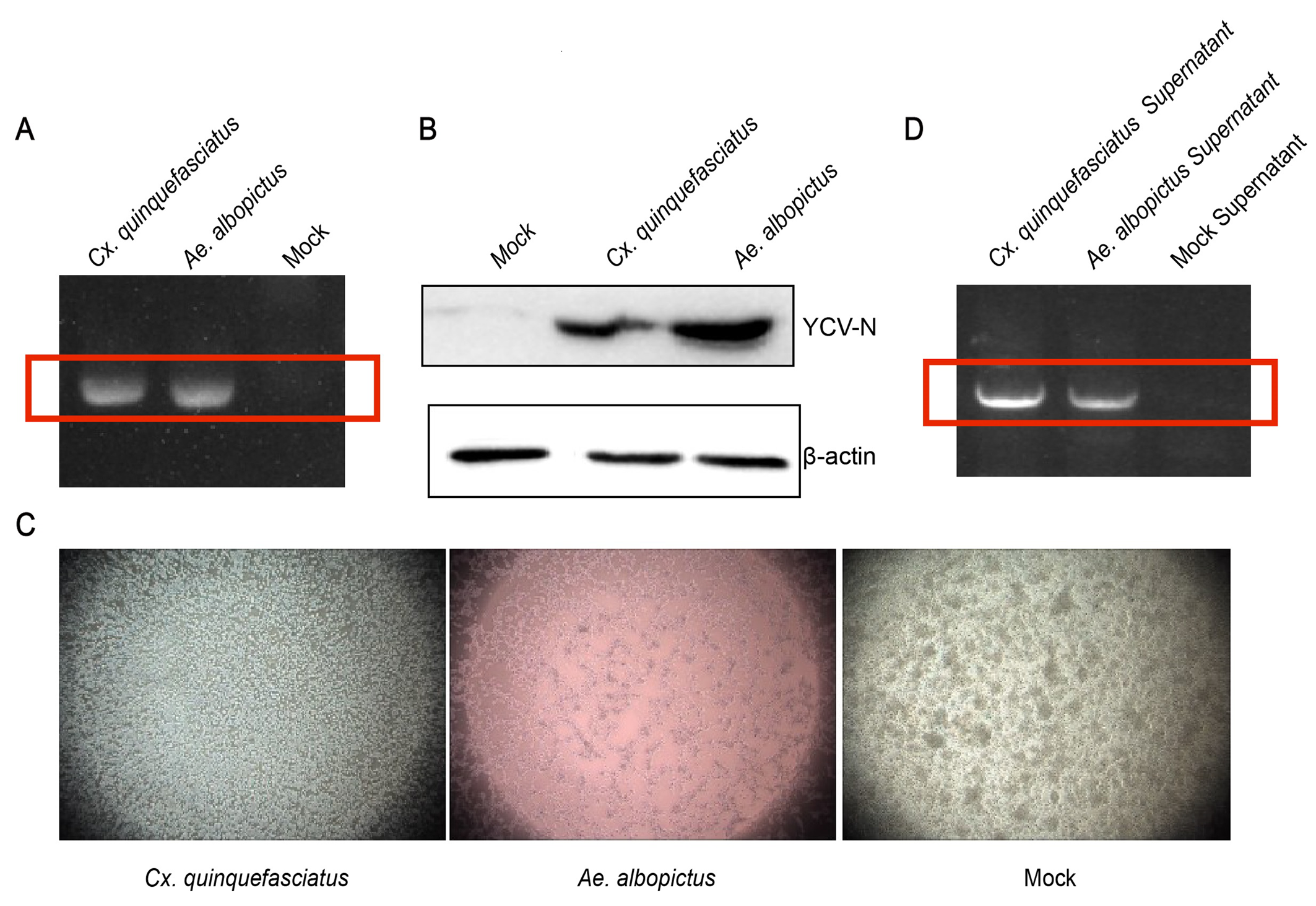

Supplement: S1 Fig — Mosquitoes were inoculated with an infectious blood meal containing a dose of 106 pfu/ml YCV. Mosquitoes were homogenized and YCV reproduction was detected by reverse transcription PCR (A) and Western blot (B) at 14 dpi. Homogenate of mosquitoes at 14 dpi was added to C6/36 cells, infectious virus particles in the whole mosquito bodies were assessed by the cytopathic effect (CPE) (C) and viruses in the supernatant of C6/36 cells were detected by reverse transcription PCR (D). (TIF) [file pntd.0008920.s001.tif]

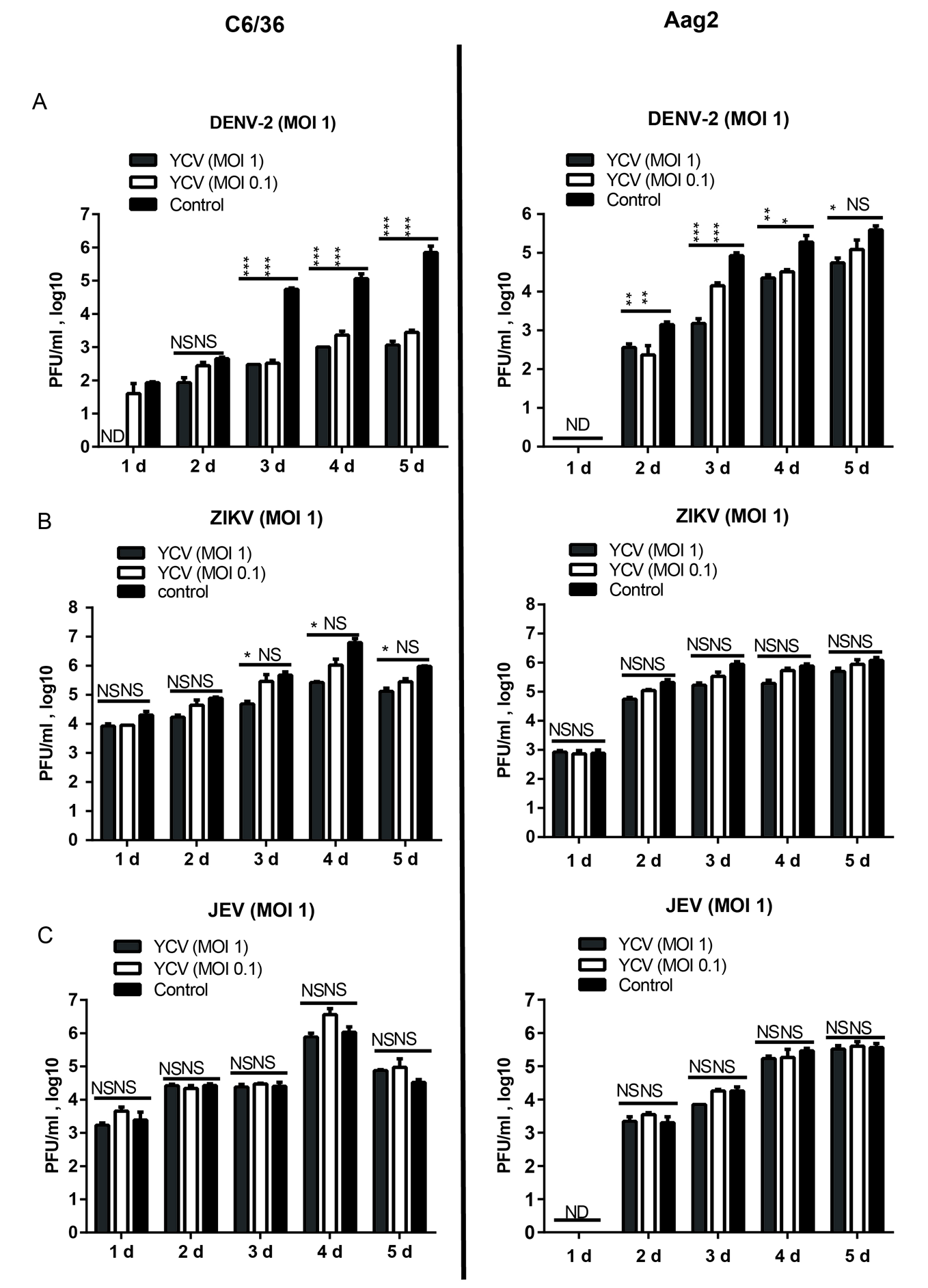

Supplement: S2 Fig — The flaviviruses DENV-2, ZIKV and JEV at MOI 1 were mixed with YCV at MOI 1 or 0.1; then, the mixture was added to C6/36 (left) or Aag2 (right) cells. (A, B, C) The virus titers during single- and coinfections were determined by the plaque assay at the indicated time points. Data are presented as the mean of three independent experiments ±SEM. The results were analyzed using the unpaired t-test. A P value of < 0.05 indicates statistical significance. P < 0.05, *; P < 0.001, ** and P < 0.001, ***. (TIF) [file pntd.0008920.s002.tif]

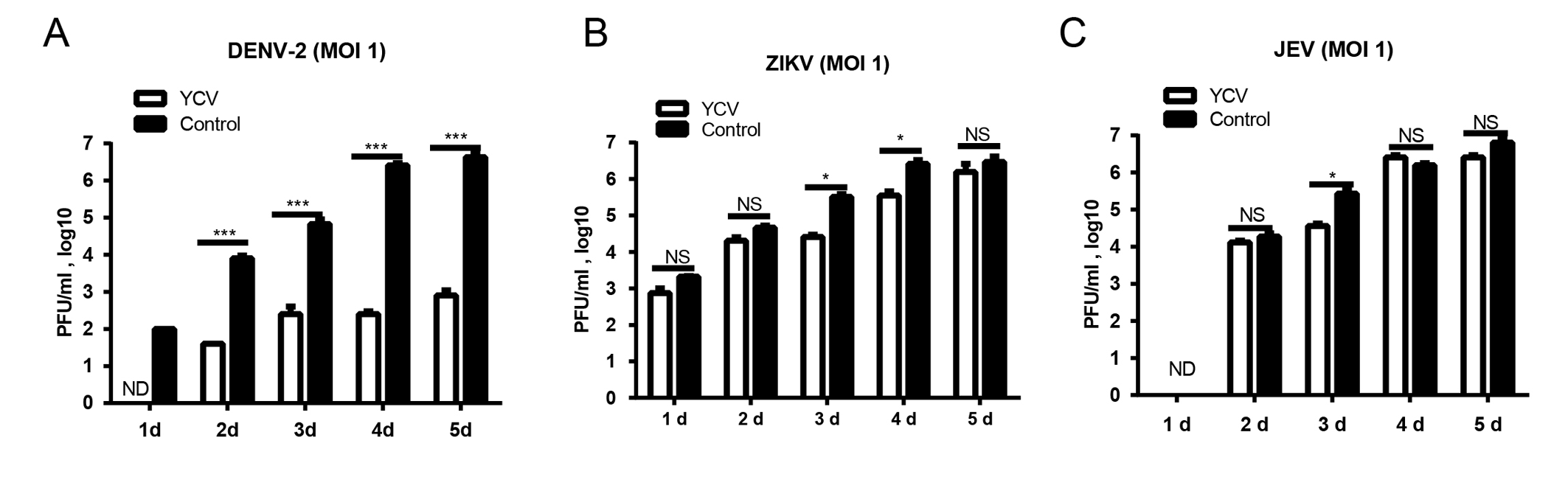

Supplement: S3 Fig — After 12 h of YCV (MOI 1) infection, C6/36 cells were infected with the flaviviruses DENV-2, ZIKV or JEV at MOI 1. (A, B, C) The virus titers during single- and coinfection were determined by the plaque assay at the indicated time points. Data are presented as the mean of three independent experiments ±SEM. The results were analyzed using unpaired t-test. A P value of < 0.05 indicates statistical significance. P < 0.05, *; P < 0.001, ** and P < 0.001, ***. (TIF) [file pntd.0008920.s003.tif]
