# Supplementary material for: Transmission competence of a new mesonivirus, Yichang virus, in mosquitoes and its interference with representative flaviviruses
Source: PLoS Negl Trop Dis. 2020 Nov 30;14(11):e0008920. doi: 10.1371/journal.pntd.0008920 (PMC7738168; doi:10.1371/journal.pntd.0008920)
Supplement: S1 Table — (DOCX) [file pntd.0008920.s004.docx]

**S1 Table. Characterization of the clean water and sewage**

| **Type** | **PH value** | **Chroma** | **Turbidity (NTU)** | **smell and taste** | **visible objects** | **COD** | **Free chlorine** | **Ca^2+^** | **Mg^2+^** |
| --- | --- | --- | --- | --- | --- | --- | --- | --- | --- |
| Clean water | 7.79 | 5 | 0.6 | no stinky odor and odorless | no | 38 | 0.1 | 40.8 | 8.72 |
| Sewage | 7.74 | 8 | 34.2 | stinky odor and odorless | yes | 47 | 0.29 | 62.1 | 16.9 |
