# Supplementary material for: Transmission competence of a new mesonivirus, Yichang virus, in mosquitoes and its interference with representative flaviviruses
Source: PLoS Negl Trop Dis. 2020 Nov 30;14(11):e0008920. doi: 10.1371/journal.pntd.0008920 (PMC7738168; doi:10.1371/journal.pntd.0008920)
Supplement: Attachment — Submitted filename: Response to reviewers comments.pdf [file pntd.0008920.s006.pdf]

Response to reviewers comments

Reviewer #1

Q1: Although the results of this study provide some biological characteristics of YCV in mosquitoes (Culex quinquefasciatus and Aedes albopictus), this manuscript only observes the research data on the co-infection of YCV and Flavivirus (Dengue virus, Zika virus, Japanese encephalitis virus) in mosquito cell lines (C6/36). This research lacks the infection results of YCV and the above Flavivirus which takes adult mosquitoes as the research object, so the research results are not enough to support the conclusion that YCV has replication interference effect on Flavivirus.

Response： Thanks for the suggestion. Since the YCV showed significantly affected the DENV-2, we have done the new experiment for YCV and DENV-2 (with titer of 3.7× 107pfu/ml and 3.7×107pfu/ml) co-infection in Aedes albopictus and the results show that YCV can reduce the infection rate and transmission rate of DENV-2 in the mosquitoes, but did not significant effect the viral load. This information has been added to the appropriate position to the section of method and result (p.15, In294-306). In addition, new table and figure (Table 2 and Fig 9) has been added.

Reviewer #2

Q1: the data is nicely presented, but can be further enriched if they can extend their discussions and thoughts on why JEV was not affected by YCV

Response： Thanks for your suggestion. YCV has been shown to impact titers for DENV-2 significantly, but no change for JEV. This could be the critical factors directly or indirectly affected by the YCV of these medically important flaviruses are viral species specificity, and further study such as direct RNA sequencing for the viruses or transcriptome analysis for the host cells can help to explain this difference. This information has been put into discussion section (p.19, In.379-382).

Q2: There was only a minor error as highlighted on lines 34-36. The authors wrote YCV is more susceptible to its hosts rather than the host being more susceptible to the virus.

Response： Thanks for your comment. We have corrected this sentence (p.2, In. 32).

Q3: The other comment is on line 320 DKV is mentioned for the first time. Its general to give the full name then the abbreviation later for ease of reading by audience who may not be familiar with the field.

Response： Thanks. The full name of DKV, Dianke virus, has been added. (p.16, ln.319)

Reviewer #3

Q1: Sample sizes and replicates of experiments are not clearly stated. More detail on the experiments conducted and what each tested need to be provided,some methods details are misplaced in the results section.

Response： We appreciate the reviewer's comment. We have added the detail information of sample sizes and the repeat times for experiments in the sections of "Mosquito rearing" (p. 5, ln. 97-99), "Mosquito infection by breeding in YCV-containing liquid" (p. 8, ln. 144-146,151-152), and "Co-infection interference in vitro" (p. 9, ln. 176-177), (p.10, In 184-186).

Q2: More detail on the experiments conducted and what each tested need to be provided

Response： Thanks for the suggestion. We have put the detail information including test content and experimental methods in the sections of "Mosquito infection by blood meal" (p. 7, ln. 138-141), "Plasmid construction and antibodies" (p. 7, ln. 128-131), and "Viral RNA detection in mosquito" (p. 9, ln. 163-165).

Q3: Some methods details are misplaced in the results section. The results section also includes methods and discussion of the results, which should be moved to the appropriate sections. The results could be condensed by referring more to the figures regarding key finding, but not describing all of the results in each of the figures in such detail.

Response： Thanks. We have moved related sentences to the appropriate section and condense the results.

Q4: In the attached track-changes version, I have made a number of grammatical and tense edits. I also left a few comments.

Rsponse： Thanks for pointing these out. We have corrected the grammatical and tense errors and response your comments in the tracking version of mauscript.

Q5: Line 94-101: are misplaced in the Introduction section

Rsponse： We have re-phrased and removed this information.

Q6: If this is a genus, it should be capitalized and italics throughout the mesonivirus.

Rsponse： We apologize for the misrepresentation and made the appropriate corrections. Yichang virus is a new species in unclassified Mesoniviridae from Virus Taxonomy: 2019 Release by ICTV. “mesonivirus” or “mesnoniviruses” means a virus group.

Q7: Line 295: unclear

Rsponse： We have modified the manuscript to include a clearer description of the degree of inhibition (p. 14, ln. 284).

Q8: Line 316: “I do not think that this is conclusive there is are a lot of other studies... Further this is unlikely to be the case as they are vertically transmitted”.

Rsponse： We appreciate the reviewer’s comment. We have modified the sentence to clarify (p. 16, ln. 312-314).
