# Supplementary material for: Transmission competence of a new mesonivirus, Yichang virus, in mosquitoes and its interference with representative flaviviruses
Source: PLoS Negl Trop Dis. 2020 Nov 30;14(11):e0008920. doi: 10.1371/journal.pntd.0008920 (PMC7738168; doi:10.1371/journal.pntd.0008920)
Supplement: Attachment — Submitted filename: PLoS NTD manuscript-6-17 - reviewer edits and comments track changes.docx [file pntd.0008920.s005.docx]

**Transmission competence of a new mesonivirus, Yichang virus, in mosquitoes and its interference with representative flaviviruses *in vitro***

Guoguo Ye^a,b^, Yujuan Wang^c^, Xiaoyun Liu^d^, Qiannan Dong^a,b^, Quanxing Cai^a^，Zhiming Yuan^a,b,^* and Han Xia^a,b,^*

**Affiliations:**

^a^Key Laboratory of Special Pathogens and Biosafety, Wuhan Institute of Virology, Center for Biosafety Mega-Science, Chinese Academy of Sciences, Wuhan, Hubei, China

^b^University of Chinese Academy of Sciences, Beijing, China

^c^Shanghai Public Health Clinical Center, Fudan University, Shanghai, China

^d^Shandong Provincial Collaborative Innovation Center for Antiviral Traditional Chinese Medicine, Shandong University of Traditional Chinese Medicine, Jinan, Shandong, China

*Correspondence: Zhiming Yuan (Email: [yzm@wh.iov.cn)](mailto:yzm@wh.iov.cn)) and Han Xia (Email: [hanxia@wh.iov.cn)](mailto:hanxia@wh.iov.cn))

**Abstract**

Advances in technology have greatly stimulated the understanding of insect-specific viruses (ISVs). Unfortunately, most of these findings are based on sequencing technology, and laboratory data are scarce on the transmission dynamics of ISVs in nature and the potential effects of these viruses on arboviruses. Mesonivirus is a class of ISVs with a wide geographical distribution. Recently, our laboratory reported the isolation of a novel strain of mesonivirus, Yichang virus (YCV), from *Culex* mosquitoes, China. In this study, the experimental infection of YCV by the oral route for adult and larvae mosquitoes, and the vertical transmission has been conducted, which suggest that YCV could adopt a mixed-mode transmission. Controlled experiments showed that the infectivity of YCV depends on the mosquito species, virus dose, and infection route. The proliferation curve and tissue distribution of YCV in *Cx. quinquefasciatus* and *Ae. albopictus* showed that YCV is more susceptible to *Ae. albopictus* and is located in the midgut. Furthermore, we also assessed the interference of YCV in the replication of flaviviruses *in vitro*. YCV significantly inhibited the proliferation of DENV-2 and ZIKV in C6/36 cell lines, while having no significant effect on JEV. Our work provides insights into the transmission of ISVs in different mosquito species during ontogeny and the potential ability of the mesonivirus YCV interfering with the replication of flaviviruses.

**Keywords:** Yichang virus, mesonivirus, mosquito, co-infection, flavivirus

**Author summary**

Mosquitoes transmit many pathogenic viruses, such as dengue virus, and Japanese encephalitis virus, Zika virus, which are great burden to public health worldwide, notably in the tropical regions. In addition, they also harbor a number of insect-specific viruses (ISVs). However, little is known about the role for the ISV in mosquito populations and their interaction with arboviruses. Yichang virus is a newly identified member of the *Mesonivirus* genus recently discovered in *Culex* mosquitoes collected from Hubei, China. In this article, we investigate the transmission competence of *Cx. quinquefasciatus* and *Ae. albopictus* for Yichang virus, and its interaction with mosquito-borne flaviviruses *in vitro*. Our study shows, for the first time, that this virus could be horizontally and vertically transmitted, and strongly inhibited the replication of the pathogenic dengue virus serotype 2 in C6/36 cells. These data provide further insight on the transmission mechanism of ISVs and how they interact with flaviviruses.

**Introduction**

For the last two decades, with advancements in high-throughput sequencing, metagenomics and intensified mosquito surveillance, a large number of insect-specific viruses (ISVs) and arboviruses have been discovered. ISVs are restricted to arthropods and are unable to replicate in vertebral cells [1], while arboviruses can be transmitted between mosquitoes and vertebrates [2]. Although these two types of viruses have different host ranges, there are many similarities in virological classification and transmission methods [2]. As arboviruses are related to many diseases of animals and humans, such as DENV, ZIKV, and JEV, their horizontal transmission and vertical transmission mechanisms are relatively clear [3-8]. However, the mode of transmission and ecological significance of the ISVs remain unknown 45 years after the discovery of ISVs by Stellar and Thomas [9, 10].

Most ISVs and arboviruses share similar genetic and structural virus particles and have a close evolutionary relationship. The relationship and interaction of the ISVs and arboviruses are complex and attract concerns worldwide. Some scholars conjecture that ISVs may be an important evolutionary source of new arboviruses [11-13] and that some ISVs may inhibit arbovirus infections in their insect hosts [10]. Moreover, the host-restricted characteristics of ISVs facilitate their development and application in biologically controlling the spread of infectious viruses [14]. In general, research on ISV transmission models and interactions with arboviruses is becoming increasingly important.

*Mesoniviridae* contains single-stranded positive-sense RNA viruses belonging to Nidovirales. So far, all members of the *Mesoniviridae* family are ISVs, with a wide geographic and population distribution [15]. Among them, Yichang virus (YCV), isolated from *Culex* mosquitoes collected from Hubei, China, has the largest genome ever discovered [16]. Due to the extensive geographic distribution and host range of *Mesoniviridae*, as well as their potential as biological control agents, more studies are needed to better understand arthropod-restricted virus maintenance in nature and the potential impact on arbovirus infection.

In this study, we investigated horizontal transmission through an oral virus challenge at the adult stage and feeding with virus-contaminated water during the larval stage and vertical transmission of YCV in *Culex quinquefasciatus* and *Aedes albopictus*. The effects of virus titers, breeding water and mosquito species on YCV infectivity were also confirmed. The proliferation and tissue distribution of YCV in *Cx. quinquefasciatus* and *Ae. albopictus* suggested that YCV was midgut restricted and that *Ae. albopictus* may be a more efficient host of YCV than *Cx. quinquefasciatus*. Furthermore, the coinfection experiments with YCV and flaviviruses showed that the presence of YCV inhibited DENV-2 and ZIKV replication, but no significant effect on JEV replication was observed *in vitro*. These laboratory-based studies deepen our understanding of the transmission mechanism of ISVs and provide important data for the implementation of ISVs in vector-borne virus control.

**Materials and methods**

***Mosquito rearing***

Both *Cx. quinquefasciatus* and *Ae. albopictus* were maintained for more than 10 years in our laboratory. Larvae and adults were maintained at 27±1˚C with a 12:12 light:dark cycle and 70% relative humidity. Adult mosquitoes were provided with 10% ad libitum glucose solution. Defibrillated horse blood (Shanghai Yuanye Biological Technology Co., Ltd. Shanghai, China) was provided through Parafilm using the Hemotek ® membrane blood feeding system (Hemotek, Lancaster, UK). *Culex quinquefasciatus* and *Ae. albopictus* parent females oviposited after the YCV blood meal. Mosquito breeding was the same as above and appropriate numbers of F1 generation mosquitoes were tested at different stages: egg, larval, pupa and adult.

***Virus strain***

The YCV strain used in this study was originally isolated in a culture of C6/36 cells inoculated with homogenized *Culex* mosquitoes from the Yichang area of Hubei [16]. C6/36 cells were cultured as monolayers in T75 flasks at 28˚C in RPMI medium (Gibco) supplemented with 10% FBS, 2% tryptose phosphate broth (Gibco); YCV was grown in C6/36 cells for viral production. DENV-2 (strain TSV01), ZIKV strain (SZ-WIV01) and JEV (strain SA14) were kindly provided by Professor Bo Zhang (Wuhan Institute of Virology, Chinese Academy of Sciences, Wuhan, China).

***Plaque assay***

BHK-21 cells in 24-well plates were infected with 10-fold serial dilutions of viruses for 1 h at 37°C. The cell monolayers were overlaid with 1% Aquacide II (Calbiochem) in DMEM containing 2% FBS and incubated at 37°C for 4 days. Monolayer cells were fixed with 3.7% formaldehyde and stained with 1% crystal violet to visualize plaques.

***Plasmid construction and antibodies***

The cDNA encoding the full-length YCV nucleotide capsid protein (abbreviation YCV-N) was coded by ORF2b cloned into vector pet28a using primers (Forward-1 5′-atgccaggacgcaccaacaca-3′ and Reverse-1 5′-ggggtcaacagtaataacataatcagcag-3′). Recombinant protein expression was induced in the *E. coli* BL21 DE3 strain using 1 M IPTG for inclusion bodies, separated, and purified by SDS-PAGE. The protein was used for subsequent polyclonal antibody preparation in the laboratory at the Animal Center, Wuhan Institute of Virology, Chinese Academy of Sciences, following standard animal procedures. A 178-bp fragment between nucleotides 154 and 331 of YCV was amplified by specific primers (Forward-2: 5′-ccaggtttgagcgaacaggt-3′; and Reverse-2: 5′-tcggggtgcggttaaaagtg-3′) and cloned into the pet28a vector for constructing the standard.

***Infectious blood meal***

Approximately 25 female mosquitoes were starved for at least 16 h at 5-7 d post-eclosion. The mosquitoes were fed defibrillated horse blood (Shanghai Yuanye Biological Technology Co., Ltd. Shanghai, China) mixed with YCV solution at 1:1 via Parafilm using the Hemotek ® membrane blood feeding system (Hemotek, Lancaster, UK). Mosquitoes were allowed to feed for 1 h *ad libitum* in light conditions, at 24˚C and 70% relative humidity (RH). Mosquitoes were anesthetized with ice and fully engorged females were selected and maintained at 28˚C. The glucose solution was refreshed every 1–2 d until 14 days post infection (dpi).

***Mosquito infection by breeding in YCV-containing liquid***

Water (clean water and sewage from near 52 Hongshan Side Road, Wuhan, China) (Table S1) was mixed with YCV supernatant from infected C6/36 cells. The mixture was used for breeding the 3-4 instar larvae of *Cx. quinquefasciatus* and *Ae. albopictus*. After a 1- to 2-day exposure to the mixture, the larvae were transferred to a container with fresh water until eclosion. The emerging mosquitoes were reared for an additional 8 days for viral detection.

***Virus assay of mosquitoes***

Total RNA was isolated using RNAiso Plus reagent (Takara, Dalian, Japan) according to the manufacturer's protocol and dissolved in 60 µL RNase-free water. [Real-time PCR](https://www.sciencedirect.com/topics/medicine-and-dentistry/real-time-polymerase-chain-reaction) was performed using the One Step SYBR Prime ScriptTM PLUS RT-PCR Kit (TaKaRa, Japan) on a MyiQTM2 Optics Module (Bio-Rad, USA). The primers were as follows: YCV detection primers are the same as the standard control plasmid construction described above. AalRPS17 was used as the internal control for qRT-PCR. The primer sequences for AalRPS17 primers were forward 5′- acgtagttgtctctctgcgctc-3′ and reverse 5′- cgcttggtttcgtgacacatc-3′. A standard curve (linear curve slope –3.5715, Y intercept 41.552, R2=0.9988, amplification efficiency 90.546) was generated from a range of serial 10-fold dilutions of the plasmid (1.8 ×10^2^-1.8 ×10^7^ copies/μl) and was used to normalize the genomic copies.

***Western blotting***

Heads and midguts from 5-10 mosquitoes and whole bodies of 5-10 mosquitoes at 14 days post-feeding of the virus blood meal were washed three times with cold PBS. Total protein was extracted by homogenizing samples and lysing in RIPA buffer (Sangon, Shanghai, China) for 30 min at 4°C. Protein concentration was determined using the BSA protein Assay Kit (Takara). YCV was detected with a mouse polyclonal antibody produced by immunizing mice with protein YCV-N. Actin was used as an internal control and detected with a mouse pan-actin antibody (clone C4; Millipore, Billerica, MA).

***Superinfection interference* in vivo**

**Simultaneous infections:** Before infection, YCV was mixed with DENV-2, ZIKV or JEV according to the specified multiplicity of infection (MOI) and then incubated with C6/36 or Aag2 cells for 1 hour at 28°C with 5% CO^2^. After incubation, cells were rinsed once with PBS and 1 mL of complete media was added. Cells were incubated for 5 days at 28°C with 5% CO^2^. Supernatant was collected at the indicated time points for the plaque assay to determine infectious virus.

**Sequential infections:** The method of sequential infections was similar to that for simultaneous infection, but YCV (MOI 1) was incubated with C6/36 after 12 h, and then the C6/36 cells containing YCV were incubated with DENV-2, ZIKV or JEV. Specific methods and conditions are similar to those for simultaneous infections, as described above.

***Statistical analysis***

Statistical analysis (Student's t-test) was performed using R.3.5.1 (https://www.r-project.org/). In all tests, the data represent the mean ± SEM. The results were analyzed using the unpaired t-test with statistical significance alpha (α) levels denoted as P < 0.05, *; P < 0.001, ** and P < 0.001, ***.

**Results**

***Mosquitoes are permissive to oral YCV infection in a dose-dependent manner***

To detect the ability of YCV to spread through the oral route, a mosquito blood-sucking infection experiment was performed. YCV reproduction was detected in total RNA from the whole mosquito bodies of *Cx. quinquefasciatus* (26.9%) and *Ae. albopictus* (45.5%) by RT-PCR (Table 1, S1 Fig). Western blot results showed that the YCV structural protein N was expressed in both *Cx. quinquefasciatus* and *Ae. albopictus* (S1 Fig). We also investigated the infectious activity of these viruses in C6/36 cells. In comparison to the control, 17.6% and 33.3% of the homogenates of *Cx. quinquefasciatus* and *Ae. albopictus* resulted in cytopathy in C6/36 cells, respectively (Table 1, S1 Fig), and all the supernatants from positive cells contained YCV RNA, confirmed by RT-PCR (S1 Fig). All the results indicate that YCV could infect *Cx. quinquefasciatus* and *Ae. albopictus* adults through the oral route.

The infectivity effect factor of the virus dose was verified with a series of YCV titers (1×10^2^, 1×10^3^, 1×10^4^, 1×10^5^ and 1×10^6^ pfu/ml) mixed with defibrillated horse blood (50% v/v) to infect mosquitoes. YCV infected both *Cx. quinquefasciatus* and *Ae. albopictus* at 1×10^6^ pfu/ml by blood-meal feeding, with a stronger infectivity in *Ae. albopictus*. The average copy number of YCV in *Ae. albopictus* (3.0×10^7^ copies/μl) was 10-fold higher than in *Cx. quinquefasciatus* (4.07×10^6^ copies/μl) at 10^6^ pfu/ml (Fig 1A), and the infection rate in *Ae. albopictus* (43%) was also higher than that in *Cx. quinquefasciatus* (31%) (Fig 1B). However, with lower titers of YCV, its infectivity to both *Cx. quinquefasciatus* and *Ae. albopictus* decreased dramatically. The 1×10^5^ pfu/ml virus infection resulted in 8.7×10^4^ copies/μl in *Ae. albopictus* and 3.16×10^4^ copies/μl in *Cx. quinquefasciatus* (Fig 1A), corresponding to 8.9% and 8.5% infection rates, respectively (Fig 1B). When the infective YCV titer was less than 1×10^4^ pfu/ml, no infection of the two mosquitoes was observed (Fig 1A and 1B). These results are consistent with that of Vasilakis, N. and Nasar, F [17, 18], revealing that only high titers of YCV can infect *Cx. quinquefasciatus* and *Ae. albopictus*.

***Mosquitoes could acquire YCV in an aquatic environment, and particles enhance virus acquisition ability***

To understand whether YCV can be acquired by mosquito larvae while breeding under aquatic conditions, the effect of breeding water on the ability of the virus to infect larvae was investigated (S2 Table). The number of YCV copies in *Cx. quinquefasciatus* were not significantly different when mosquitoes were bred in sewage (1.34×10^5^ copies/μl) or clean water (1.49×10^5^ copies/μl) containing 10^5^ pfu/ml of YCV (Fig 2A), while the positive rate in sewage (10.66%) was slightly higher than that in clean water (5.66%) (Fig 2B). Interestingly, at the treatment concentrations of 10^3^ pfu/ml and 10^4^ pfu/ml, the virus copies were much higher for *Cx. quinquefasciatus* (1.09×10^4^ and 4.46×10^4^ copies/μl) in the sewage than in clean water (15.89×10^0^ and 4.16×10^3^ copies/μl) (Fig 2A), but the positive rate was low (<3％) (Fig 2B). Similarly, the infection pattern of YCV on *Ae. albopictus* was comparable to that of *Cx. quinquefasciatus* in the sewage and clean water (Fig 2A and 2B). The results show that the infectivity present in larvae incubated in clean water or sewage was dose-dependent; *Ae. albopictus* was more susceptible to YCV than *Cx. quinquefasciatus;* and YCV had a stronger infection ability in sewage than in clean water for both *Ae. albopictus* and *Cx. quinquefasciatus*.

Next, we examined whether there are any factors in the sewage that affect the YCV infection capacity. Sewage water, sewage water filtered at 0.22 µm to remove particles, clean water, and clean water with the addition of particles derived from sewage were used for mosquito breeding. The average number of copies increased by approximately 10^2^-fold in the clean water with added particles, with a significantly increased infection rate from 2.17% to 13.2% in *Cx. quinquefasciatus* (Fig 3A and 3B). In contrast, the average copies of YCV in *Cx. quinquefasciatus* were reduced by approximately 10^4^-fold with a lower infection rate of 1.43% in the filtered sewage compared to the sewage treatment, which had an infection rate of 4.51%. Furthermore, along with the restoration of the particles in sewage, the average YCV copy number in *Ae. albopictus* increased by 10-fold, and the infection rate also increased from 16.05% to 39.68%. However, the average YCV copy number in *Ae. albopictus* decreased by approximately 10^2^-fold when incubated in filtered sewage compared to clean water, and the infection rate also decreased from 34.65% to 15.04% (Fig 3A and 3B). This result suggests that the particles in water affect the infectivity of YCV to mosquitoes, which might be related to the viral stability, but remains to be further investigated.

***YCV vertical transmission in mosquitoes***

To investigate the vertical transmission of YCV, the presence of YCV in different developmental stages of the F1 generation of blood meal-fed mosquitoes was detected. YCV copies were low among the eggs, larvae, pupae and adults of *Cx. quinquefasciatus*, and the RNA copies decreased in order (Fig 4A). Adults had the lowest positive rate (10%), while there were no marked differences between larvae (18.05%) and pupae (21.13%) (Fig 4B). However, higher virus copies and positivity rates were detected in adults of the *Ae. albopictus* F1 generation than in larvae and pupae (Fig 4A and 4B). Although vertical transmission is the primary way to maintain and transmit ISVs in nature, the lower vertical transmission efficiency of YCV suggests that vertical transmission may only be one of its transmission methods. We infer that the maintenance and transmission of YCV in nature may be the result of a combination of multiple transmission methods.

***Proliferation and distribution of YCV in mosquitoes***

Mosquitoes ingesting the infectious blood meal were tested for YCV gene copies at each time point. RT-qPCR revealed that all *Ae. albopictus* were YCV virus positive at 0 dpi (Fig 5 B); the YCV copies increased steadily until 7 dpi and then maintained a stable level in later periods (p>0.05) (Fig 5A). On the other hand, the virus copies in *Cx. quinquefasciatus* steadily decreased from 7.5×10^5^ copies/μl to 8.8×10^3^ copies/μl in the first three days, but increased to 4.7×10^6^ copies/μl at 7 dpi, and then maintained at the same level in later mosquito development stages (p＞0.05) (Fig 5A). It is clear that not only the higher infection rate but also higher viral copies at dpi 4, 7, 10, and 14, were observed in *Ae. Albopictus*, suggesting that *Ae. albopictus* may be a better host for YCV than *Cx. quinquefasciatus*.

We detected the presence of YCV nucleic acid and structural protein N in the head and midgut of the infected *Cx. quinquefasciatus* and *Ae. albopictus*. Western blot results showed that the YCV structural protein N can be detected in the midgut of *Cx. quinquefasciatus* and *Ae. albopictus*, but not in the head (Fig 6A). YCV nucleic acid was detected in the midgut, with higher RNA expression in *Ae. albopictus* than in *Cx. quinquefasciatus*, but not in the head of both mosquitoes (Fig 6B). We conclude that the midgut is the main target organ for the infection and multiplication of YCV, and *Ae. albopictus* may be a more susceptible host for YCV infection.

***YCV inhibits replication of pathogenic flaviviruses* in vitro**

To examine the effects of the persistence of YCV on the replication of pathogenic flaviviruses, mosquito C6/36 and Aag2 cells were simultaneously or sequentially infected with YCV and three flaviviruses (DENV-2, ZIKV and JEV). As shown in Fig 7, the presence of YCV significantly inhibits DENV-2 replication in C6/36 cells and Aag2 cells, with replications of 10^2^, ~10^3-^, and 10 ~10^2-^fold lower in C6/36 and Aag2 cells when DENV-2 is co-inoculated with YCV than with DENV-2 infection alone, respectively (Fig 7A, S3 Fig A). In addition, a 10-fold lower ZIKV replication was observed when ZIKV (MOI = 0.1 and MOI = 0.1) was coinoculated with YCV (MOI = 0.1 and MOI = 1) than under ZIKV inoculation alone in the C6/36 cells at 3, 4, and 5 dpi (Fig 7 B, S3 Fig B). However, no synergism or inhibition of YCV on JEV replication in the two cell lines was observed for any of the different coinoculation combinations (YCV MOI = 1/JEV MOI = 1, YCV MOI = 1/JEVMOI=0.1, YCV MOI = 0.1/JEVMOI = 0.1 and YCV MOI = 0.1/JEVMOI = 0.1) were used (Fig 7C, S3 Fig C). Furthermore, the effects of the infection sequence order of YCV and flaviviruses on the replication of pathogenic flaviviruses in C6/36 cells were also studied. The replication of DENV-2 and ZIKV is 10^4^-fold and 10^1^-fold lower, respectively, in C6/36 cells preinfected with YCV for 12 h in comparison with the control, whether the infection titer of the DENV-2 and ZIKV was high (MOI=1) or low (MOI=0.1) (Fig 8A and 8B, S4 Fig A and B). Not surprisingly, the preinfection of JEV had no effect on the replication of JEV in cells (Fig 8C, S4 Fig C).

**Discussion**

The results presented here indicate that the insect-specific virus YCV may be transmitted and maintained in the environment using a complex transmission and maintenance model, including virus infection and transmission in the larval and adult stages, as well as localization in mosquito tissues [19]. The localization of YCV in midgut tissues was consistent with other insect-specific flavivirus (ISFs), which appear to be restricted to the midgut of their mosquito hosts [20, 21]. Although YCV can effectively infect *Cx. quinquefasciatus* and *Ae. albopictus* at 10^6^ pfu/ml by the oral route, its infection ability is significantly reduced and even vanishes with reducing titers, suggesting that oral susceptibility can vary greatly or is even restricted in some mosquito species. However, recent studies using DKV showed conflicting results. *Culex quinquefasciatus*, *Cx. tritaeniorhynchus*, and *Ae. aegypti* were highly susceptible to infection and able to transmit DKV [22]. This may be dependent on mosquito species and the microorganisms carried by the mosquito itself.

We conducted *in vivo* growth experiments with *Cx. quinquefasciatus* and *Ae. albopictus.* The results revealed that the virus infects and replicates in the midgut and that its infection rate is much lower in *Cx. quinquefasciatus* than in *Ae. albopictus*, similar to what was observed for flaviviruses such as Bamaga virus [23]. This probably resulted from the varied midgut barrier expressed following ingestion of the virus in different mosquito species [24]. This also suggested that *Cx. quinquefasciatus* and *Ae. albopictus* express a midgut escape barrier, as none of the infected *Ae. albopictus* and *Cx. quinquefasciatus* showed a disseminated infection. A previous study showed that Eilat virus could bypass midgut barriers and spread to the salivary glands of *Ae. albopictus* and *Cx. quinquefasciatus* treated with thoracic injection [17]. We attempted to address the ability of *Cx. quinquefasciatus* and *Ae. albopictus* to acquire viruses from water during the larval stage. Several studies have shown that the population of adult *Ae. aegypti* mosquitoes could be effectively infected by the virus in sewage and clean water [25]. Accumulating evidence from field surveillance convincingly shows that *A. aegypti* and *A. albopictus* tend to oviposit and breed in wastewater with low dissolved oxygen and high turbidity [26]. Our study confirmed that YCV in an aquatic environment can be acquired and subsequently transmitted by mosquitoes. Interestingly, the infectivity of YCV in sewage is higher than that in clean water, and certain ingredients in the sewage can affect viral infections. Indeed, as shown in S1 Table, we noted that the aquatic particles correlated with YCV infectivity. The difference might be caused by the divalent cations or the pH, as suggested in other studies on dsRNA virus PCV [27], DNA virus NPV [28], and Zika virus [26], or perhaps the presence of bacteria, as demonstrated in reoviruses by other authors [29]. However, the effects of divalent cations or pH on YCV infectivity in water remain to be further investigated.

Although vertical transmission has been considered an important way for ISVs to persist and disperse in nature, the relatively low proportion of YCV positive F1 generation (eggs, larvae or adult) corroborates low rates have observed for CxFV, Aedes ﬂavivirus (AeFV), Okushiri virus, and KRV [9, 30-38]. It is possible that ISVs can persist in mosquitoes for a long time, although the vertical transmission efficiency is low. Indeed, studies of *Dipteran ambidensovirus* 1 (*Cx. pipiens densovirus*) proved that the virus can persist for over 20 years in laboratory colony *Cx. pipiens* (sl.), with a low rates of vertical and transovarial transmission [39]. The low rate of YCV vertical transmission is consistent with the midgut restriction barrier expressed in the mosquito. Indeed, the virus disseminated from the midgut cells typically undergoes secondary replication in other tissues, such as fat bodies or ovaries [24]. Collectively, the vertical transmission capacity of YCV is low and horizontal transmission is also restricted to a certain extent, and mixed-mode transmission, including both horizontal and vertical transmission routes. is likely to be key for YCV to be maintained in nature, which is similar to ISFs [40, 41].

Superinfection exclusion is an important feature of ISVs. There are several studies of superinfection interference, which mostly occur between cognate viruses [13, 42, 43], as the mechanism is generally considered to be competitive inhibition. It is possible that the mechanism of superinfection exclusion also exists in addition to that of competitive inhibition. Mesoniviridae is far from the Flaviviridae, and their interactions are rarely reported. In this study, it was clear that YCV could obstruct the replication of DENV-2 and ZIKV in C6/36 cells and Aag2 cells, but had no effect on JEV. This indicated that YCV, a mesonivirus, could induce heterologous interference with several flaviviruses. Several reports suggested that flaviviruses do not interfere with viruses belonging to other families or genera in most instances [44, 45]. However, other authors report conflicting results [46, 47], which seem to agree with our *in vitro* data. A recent publication [48] demonstrated that EILV, an ISVs, induces heterologous interference with several other *Alphavirus* pathogens, suggesting that heterologous interactions can occur. Indeed, the data in our laboratory reveal that the inhibitory effect of YCV on flaviviruses was also different, since the most pronounced reduction was observed in sequential infection with DENV (10^4^-fold less), whereas in JEV, no reduction was seen in coinfections or sequential infections, suggesting that the interference was specific. Although the prevalence of arthropod-borne viruses in mosquito populations is quite low, the transmission of mosquito bites would greatly increase the epidemic risk of arbovirus-related diseases. Therefore, research on the interaction between microorganisms in mosquitoes is very important for epidemiological prediction and biological control of arboviruses.

ISVs are only maintained in insect populations, but it remains unclear how they transmit among insects. Here, we report that YCV could infect mosquitoes orally at the adult stage and breed in aquatic environments at the larva stage, with a low vertical transmission capacity. This offers laboratory evidence that mixed-mode transmission in mosquitoes, including both horizontal and vertical transmission routes, is likely to be the key for YCV maintenance in nature. The growth and tissue distribution of YCV in mosquitoes suggests that YCV is restricted to the host midgut and that *Ae. albopictus* maybe a better host for YCV than *Cx. quinquefasciatus*. Interaction data between YCV and three flaviviruses indicates that superinfection exclusion may occur not only between homologous viruses, but also between heterologous viruses, and the inhibition does not occur with all flaviviruses. It is important to understand the ecological significance of this interaction to further the understanding of mechanisms of interference between vector-borne viruses and ISVs in mosquitoes and subsequent implementation of vector-borne virus control.

**Acknowledgments**

We are thankful to Bo Zhang, Wuhan Institute of Virology, Chinese Academy of Sciences, for kindly provided DENV-2 (strain TSV01), ZIKV strain (SZ-WIV01) and JEV (strain SA14). And we would like to thank the Animal Center, Wuhan Institute of Virology, Chinese Academy of Science, for technical assistance.

**Declaration of interest statement**

The authors have no competing interest.

**Funding**

This work was supported by the Ministry of Science and Technology of the People’s Republic of China [2018ZX10101004, 2013FY113500], and the Wuhan Institute of Virology, China [WIV-135-PY2].

**Author’s Contribution**

GY performed the experiments and wrote the manuscript; QD performed the DENV-2 Plaque assay experiments; XL and YW contributed to manuscript revision; QC contributed in the mosquito rearing. ZY and HX contributed to designed the experimental, wrote and review the final manuscript.

**References**

1.	Nasar F, Gorchakov RV, Tesh RB, Weaver SC. Eilat virus host range restriction is present at multiple levels of the virus life cycle. J Virol. 2015;89(2):1404-18. PMID: 25392227.

2.	Halbach R, Junglen S, van Rij RP. Mosquito-specific and mosquito-borne viruses: evolution, infection, and host defense. Curr Opin Insect Sci. 2017;22:16-27. PMID: 28805635.

3.	Conway MJ, Colpitts TM, Fikrig E. Role of the Vector in Arbovirus Transmission. Annu Rev Virol. 2014;1(1):71-88. PMID: 26958715.

4.	Danis-Lozano R, Diaz-Gonzalez EE, Malo-Garcia IR, Rodriguez MH, Ramos-Castaneda J, Juarez-Palma L, et al. Vertical transmission of dengue virus in Aedes aegypti and its role in the epidemiological persistence of dengue in Central and Southern Mexico. Trop Med Int Health. 2019;24(11):1311-9. PMID: 31483936.

5.	da Costa CF, Dos Passos RA, Lima JBP, Roque RA, de Souza Sampaio V, Campolina TB, et al. Transovarial transmission of DENV in Aedes aegypti in the Amazon basin: a local model of xenomonitoring. Parasit Vectors. 2017;10(1):249. PMID: 28526066.

6.	Runge-Ranzinger S, Morrison AC, Manrique-Saide P, Horstick O. Zika transmission patterns: a meta-review. Trop Med Int Health. 2019;24(5):523-9. PMID: 30771269.

7.	Song B-H, Yun S-I, Woolley M, Lee Y-M. Zika virus: History, epidemiology, transmission, and clinical presentation. Journal of Neuroimmunology. 2017;308:50-64.

8.	Garcia-Nicolas O, Braun RO, Milona P, Lewandowska M, Dijkman R, Alves MP, et al. Targeting of the Nasal Mucosa by Japanese Encephalitis Virus for Non-Vector-Borne Transmission. J Virol. 2018;92(24). PMID: 30282716.

9.	Cook S, Bennett SN, Holmes EC, De Chesse R, Moureau G, de Lamballerie X. Isolation of a new strain of the flavivirus cell fusing agent virus in a natural mosquito population from Puerto Rico. J Gen Virol. 2006;87(Pt 4):735-48. PMID: 16528021.

10.	Stollar V, L.Thomas V. An agent in the Aedes aegypti cell line (Peleg) which causes fusion of Aedes albopictus cells. Virology. 1975; 64(2): 367-77.

11.	Bolling BG, Weaver SC, Tesh RB, Vasilakis N. Insect-Specific Virus Discovery: Significance for the Arbovirus Community. Viruses. 2015;7(9):4911-28. PMID: 26378568.

12.	Lutomiah JJ MC, Magambo J, Sang RC. Infection and vertical transmission of Kamiti river virus in laboratory bred Aedes aegypti mosquitoes. J Insect Sci. 2007;7:1‐7.

13.	Hobson-Peters J, Yam AW, Lu JW, Setoh YX, May FJ, Kurucz N, et al. A new insect-specific flavivirus from northern Australia suppresses replication of West Nile virus and Murray Valley encephalitis virus in co-infected mosquito cells. PLoS One. 2013;8(2):e56534. PMID: 23460804.

14.	Roundy CM AS, Rossi SL, Weaver SC, Vasilakis N. Insect-Specific Viruses A Historical Overview and Recent Developments. Adv Virus Res. 2017;98:119-146.

15.	Nga PT, Parquet Mdel C, Lauber C, Parida M, Nabeshima T, Yu F, et al. Discovery of the first insect nidovirus, a missing evolutionary link in the emergence of the largest RNA virus genomes. PLoS Pathog. 2011;7(9):e1002215. PMID: 21931546

16.	Wang Y, Xia H, Zhang B, Liu X, Yuan Z. Isolation and characterization of a novel mesonivirus from Culex mosquitoes in China. Virus Res. 2017;240:130-9. PMID: 28823942

17.	Nasar F, Haddow AD, Tesh RB, Weaver SC. Eilat virus displays a narrow mosquito vector range. Parasit Vectors. 2014;7:595. PMID: 25515341

18.	Vasilakis N, Forrester NL, Palacios G, Nasar F, Savji N, Rossi SL, et al. Negevirus: a proposed new taxon of insect-specific viruses with wide geographic distribution. J Virol. 2013;87(5):2475-88. PMID: 23255793

19.	Agboli E, Leggewie M, Altinli M, Schnettler E. Mosquito-Specific Viruses-Transmission and Interaction. Viruses. 2019;11(9). PMID: 31533367

20.	AMG C, J H-P, H B-O, AF vdH, S H-M, WK C, et al. A New Clade of Insect-Specific Flaviviruses from Australian Anopheles Mosquitoes Displays Species-Specific Host Restriction. mSphere. 2017

21.	Hall-Mendelin S, McLean BJ, Bielefeldt-Ohmann H, Hobson-Peters J, Hall RA, van den Hurk AF. The insect-specific Palm Creek virus modulates West Nile virus infection in and transmission by Australian mosquitoes. Parasit Vectors. 2016;9(1):414. PMID: 27457250

22.	Gaye A, Diagne MM, Ndiaye EH, Dior Ndione MH, Faye M, Talla C, et al. Vector competence of anthropophilic mosquitoes for a new mesonivirus in Senegal. Emerg Microbes Infect. 2020;9(1):496-504. PMID: 32106784

23.	Colmant AMG, Hall-Mendelin S, Ritchie SA, Bielefeldt-Ohmann H, Harrison JJ, Newton ND, et al. The recently identified flavivirus Bamaga virus is transmitted horizontally by Culex mosquitoes and interferes with West Nile virus replication in vitro and transmission in vivo. PLoS Negl Trop Dis. 2018;12(10):e0006886. PMID: 30356234.

24.	Kuno G, Chang GJ. Biological transmission of arboviruses: reexamination of and new insights into components, mechanisms, and unique traits as well as their evolutionary trends. Clin Microbiol Rev. 2005;18(4):608-37. PMID: 16223950.

25.	Burke R, Barrera R, Lewis M, Kluchinsky T, Claborn D. Septic tanks as larval habitats for the mosquitoes Aedes aegypti and Culex quinquefasciatus in Playa-Playita, Puerto Rico. Med Vet Entomol. 2010;24(2):117-23. PMID: 20374477.

26.	Du S, Liu Y, Liu J, Zhao J, Champagne C, Tong L, et al. Aedes mosquitoes acquire and transmit Zika virus by breeding in contaminated aquatic environments. Nat Commun. 2019;10(1):1324. PMID: 30902991

27.	Green TB, Shapiro A, White S, Rao S, Mertens PP, Carner G, et al. Molecular and biological characterization of a Cypovirus from the mosquito Culex restuans. J Invertebr Pathol. 2006;91(1):27-34. PMID: 16376932.

28.	Shapiro A, Green T, Rao S, White S, Carner G, Mertens PP, et al. Morphological and molecular characterization of a Cypovirus (Reoviridae) from the mosquito Uranotaenia sapphirina (Diptera: Culicidae). J Virol. 2005;79(15):9430-8. PMID: 16014906

29.	Berger AK, Yi H, Kearns DB, Mainou BA. Bacteria and bacterial envelope components enhance mammalian reovirus thermostability. PLoS Pathog. 2017;13(12):e1006768. PMID: 29211815

30.	Ajamma YU, Onchuru TO, Ouso DO, Omondi D, Masiga DK, Villinger J. Vertical transmission of naturally occurring Bunyamwera and insect-specific flavivirus infections in mosquitoes from islands and mainland shores of Lakes Victoria and Baringo in Kenya. PLoS Negl Trop Dis. 2018;12(11):e0006949. PMID: 30452443

31.	Sang RC, Gichogo A, Gachoya J, Dunster MD, Ofula V, Hunt AR, et al. Isolation of a new flavivirus related to cell fusing agent virus (CFAV) from field-collected flood-water Aedes mosquitoes sampled from a dambo in central Kenya. Arch Virol. 2003;148(6):1085-93. PMID: 12756616.

32.	Misencik MJ, Grubaugh ND, Andreadis TG, Ebel GD, Armstrong PM. Isolation of a Novel Insect-Specific Flavivirus from Culiseta melanura in the Northeastern United States. Vector Borne Zoonotic Dis. 2016;16(3):181-90. PMID: 26807512

33.	Auguste AJ, Kaelber JT, Fokam EB, Guzman H, Carrington CV, Erasmus JH, et al. A newly isolated reovirus has the simplest genomic and structural organization of any reovirus. J Virol. 2015;89(1):676-87. PMID: 25355879

34.	Bolling BG, Eisen L, Moore CG, Blair CD. Insect-specific flaviviruses from Culex mosquitoes in Colorado, with evidence of vertical transmission. Am J Trop Med Hyg. 2011;85(1):169-77. PMID: 21734144

35.	Fauver JR, Grubaugh ND, Krajacich BJ, Weger-Lucarelli J, Lakin SM, Fakoli LS, 3rd, et al. West African Anopheles gambiae mosquitoes harbor a taxonomically diverse virome including new insect-specific flaviviruses, mononegaviruses, and totiviruses. Virology. 2016;498:288-99. PMID: 27639161.

36.	Haddow AD, Guzman H, Popov VL, Wood TG, Widen SG, Haddow AD, et al. First isolation of Aedes flavivirus in the Western Hemisphere and evidence of vertical transmission in the mosquito Aedes (Stegomyia) albopictus (Diptera: Culicidae). Virology. 2013;440(2):134-9. PMID: 23582303.

37.	Yamanaka A, Thongrungkiat S, Ramasoota P, Konishi E. Genetic and evolutionary analysis of cell-fusing agent virus based on Thai strains isolated in 2008 and 2012. Infect Genet Evol. 2013;19:188-94. PMID: 23871775.

38.	Kawakami K, Kurnia YW, Fujita R, Ito T, Isawa H, Asano S, et al. Characterization of a novel negevirus isolated from Aedes larvae collected in a subarctic region of Japan. Arch Virol. 2016;161(4):801-9. PMID: 26687585.

39.	Altinli M, Soms J, Ravallec M, Justy F, Bonneau M, Weill M, et al. Sharing cells with Wolbachia: the transovarian vertical transmission of Culex pipiens densovirus. Environ Microbiol. 2018. PMID: 30585387.

40.	Kim DY, Guzman H, Bueno R, Jr., Dennett JA, Auguste AJ, Carrington CV, et al. Characterization of Culex Flavivirus (Flaviviridae) strains isolated from mosquitoes in the United States and Trinidad. Virology. 2009;386(1):154-9. PMID: 19193389.

41.	Obara-Nagoya M, Yamauchi T, Watanabe M, Hasegawa S, Iwai-Itamochi M, Horimoto E, et al. Ecological and genetic analyses of the complete genomes of Culex flavivirus strains isolated from Culex tritaeniorhynchus and Culex pipiens (Diptera: Culicidae) group mosquitoes. J Med Entomol. 2013;50(2):300-9. PMID: 23540117.

42.	Bolling BG, Olea-Popelka FJ, Eisen L, Moore CG, Blair CD. Transmission dynamics of an insect-specific flavivirus in a naturally infected Culex pipiens laboratory colony and effects of co-infection on vector competence for West Nile virus. Virology. 2012;427(2):90-7. PMID: 22425062

43.	Kenney JL, Solberg OD, Langevin SA, Brault AC. Characterization of a novel insect-specific flavivirus from Brazil: potential for inhibition of infection of arthropod cells with medically important flaviviruses. J Gen Virol. 2014;95(Pt 12):2796-808. PMID: 25146007

44.	Kanthong N, Khemnu N, Sriurairatana S, Pattanakitsakul SN, Malasit P, Flegel TW. Mosquito cells accommodate balanced, persistent co-infections with a densovirus and Dengue virus. Dev Comp Immunol. 2008;32(9):1063-75. PMID: 18397805.

45.	Kanthong N, Khemnu N, Pattanakitsakul SN, Malasit P, Flegel TW. Persistent, triple-virus co-infections in mosquito cells. BMC Microbiol. 2010;10:14. PMID: 20089150

46.	Burivong P, Pattanakitsakul SN, Thongrungkiat S, Malasit P, Flegel TW. Markedly reduced severity of Dengue virus infection in mosquito cell cultures persistently infected with Aedes albopictus densovirus (AalDNV). Virology. 2004;329(2):261-9. PMID: 15518806.

47.	Muturi EJ, Bara J. Sindbis virus interferes with dengue 4 virus replication and its potential transmission by Aedes albopictus. Parasit Vectors. 2015;8:65. PMID: 25633862

48.	Nasar F, Erasmus JH, Haddow AD, Tesh RB, Weaver SC. Eilat virus induces both homologous and heterologous interference. Virology. 2015;484:51-8. PMID: 26068885

Table 1. Rate of YCV Infection for *Cx. quinquefasciatus* and *Ae. albopictus*

| **Species** | **Blood meal titer (pfu/ml)** | **No. YCV RNA positive/No. engorged** | **No. CPE observed/No. engorged** |
| --- | --- | --- | --- |
| *Cx. quinquefasciatus* | 10^6^ | (7/26) 26.9% | (3/17) 17.6% |
| *Ae. albopictus* | 10^6^ | (10/22) 45.5% | (4/12) 33.3% |

**Figure Captions**

**Fig 1.** **Infectivity and infection rates of *Cx. quinquefasciatus* and *Ae. albopictus* infected with different doses of YCV**. Mosquitoes were inoculated with an infectious blood meal containing a dose of 10^6^, 10^5^, 10^4^, 10^3^ or 10^2^ pfu/ml YCV. (A) Ingested virus titers of *Cx. quinquefasciatus* and *Ae. albopictus* by qRT- PCR at 14 dpi. Each dot represents one mosquito body; the results are expressed as the mean ± SEM. (B) Infection rates of *Cx. quinquefasciatus* and *Ae. albopictus* at 14 dpi presented as the percentage of the total number of engorged mosquitoes. Shown are the mean percentages from three independent replicates. Error bars show the standard error of the mean. Sample size ranged between 10–15 female mosquitoes per treatment. The results were analyzed using the unpaired t-test. A P value of < 0.05 indicates statistical significance. P < 0.05, *; P < 0.001, ** and P < 0.001, ***.

**Fig 2. YCV in an aquatic environment can be acquired by *Cx. quinquefasciatus* and *Ae. Albopictus.*** *Cx. quinquefasciatus* and *Ae. albopictus* were bred in clean water or sewage with a serial YCV titration (10^5^, 10^4^, 10^3^ and 10^2^ pfu/ml). (A) The emerging adults were reared for an additional 8 days for YCV detection by qRT-PCR. Each dot represents one mosquito body; the results are expressed as the mean ± SEM. (B) Infection rates of *Cx. quinquefasciatus* and *Ae. albopictus* are represented as the ratios of mosquito infection. The results are expressed as the mean ± SEM. The results were analyzed using the unpaired t-test. A P value of < 0.05 indicates statistical significance. P < 0.05, *; P < 0.001, ** and P < 0.001, ***.

**Fig 3. Particulate matter in sewage enhances YCV infectivity of *Cx. quinquefasciatus* and *Ae. albopictus*.** Larvae (3^rd^-4^th^ instar) were bred in clean water, clean water+particles, sewage, or sewage-particles with YCV (final YCV titer was 10^4^ pfu/ml). (A) YCV RNA titers of *Cx. quinquefasciatus* (left) and *Ae. albopictu*s (right) were detected by QRT-PCR 8 days after emergence. One dot represents one mosquito. The results are expressed as the mean ± SEM. (B) Infection rates. The results were analyzed using the unpaired t-test. A P value of < 0.05 indicates statistical significance. P < 0.05, *; P < 0.001, ** and P < 0.001, ***.

**Fig 4. Viral load of YCV in different life stages of the F1 generation.** After the infected *Cx. quinquefasciatus* and *Ae. albopictus* parent females had oviposited, egg papers were hatched in deoxygenated water. (A) Offspring were reared and YCV RNA copies were detected by QRT-PCR in different life stages (egg, larva, pupa and adult). One dot represents one mosquito. The results are expressed as the mean ± SEM. (B) Positive rate. The results are expressed as the mean ± SEM. The results were analyzed using the unpaired t-test. A P value of < 0.05 indicates statistical significance. P < 0.05, *; P < 0.001, ** and P < 0.001, ***

**Fig 5**. **Virus reproduction and infection rates for YCV in *Cx. quinquefasciatus* and *Ae. albopictus*.** The mosquitoes were fed a mixture containing horse blood (50% v/v) with 10^6^ pfu/ml YCV. (A) YCV virus RNA copies in the whole mosquito bodies were detected by QRT- PCR at 0 dpi, 3 dpi, 7 dpi, 11 dpi, and 14 dpi. The results are expressed as the mean ± SEM. (B) Infection rates of *Cx. quinquefasciatus* and *Ae. albopictus* at the indicated time points presented as the percentage of the total number of engorged mosquitoes. Shown are the mean percentages from three independent replicates. Error bars show the standard error of the mean. Sample size ranged from 10–15 female mosquitoes per treatment. The results were analyzed using the unpaired t-test. A P value of < 0.05 indicates statistical significance. P < 0.05, *; P < 0.001, ** and P < 0.001, ***.

**Fig 6. Assessing the infectivity of midgut and head of *Cx. quinquefasciatus* and *Ae. albopictus*.** The mosquitoes were fed a mixture containing horse blood (50% v/v) with 106 pfu/ml YCV titration. Viral protein and RNA of the midgut and head of Cx. quinquefasciatus and Ae. albopictus were detected by Western blot (A) and quantitative reverse transcription PCR (B) at 14 dpi. Data are presented as the mean ± SEM. The sample size ranged from 10–15 female mosquitoes per treatment. The results were analyzed using the unpaired t-test. A P value of < 0.05 indicates statistical significance. P < 0.05, *; P < 0.001, ** and P < 0.001, ***.

**Fig 7. YCV effects the growth of DENV-2, ZIKV and JEV in C6/36 and Aag2 cells during single- and coinfections of simultaneous infections.** The flaviviruses DENV-2, ZIKV and JEV at MOI=0.1 were mixed with YCV at MOI=1 or 0.1; the mixture was then added to C6/36 (left) or Aag2 (right) cells. (A, B, C) The virus titers during single- and coinfection were determined by plaque assays at the indicated time points. Data are presented as the mean of three independent experiments ±SEM. The results were analyzed using the unpaired t-test. A P value of < 0.05 indicates statistical significance. P < 0.05, *; P < 0.001, ** and P < 0.001, ***.

**Fig 8. YCV effects the growth of DENV-2, ZIKV and JEV in C6/36 cells during single- and coinfections of sequential infections**. After 12 h of YCV (MOI=1) infection, C6/36 cells were infected with the flaviviruses DENV-2, ZIKV or JEV at MOI=0.1. (A, B, C) The virus titers during single- and coinfection were determined by plaque assays at the indicated time points. Data are presented as the mean of three independent experiments ±SEM. The results were analyzed using the unpaired t-test. A P value of < 0.05 indicates statistical significance. P < 0.05, *; P < 0.001, ** and P < 0.001, ***.

**Supporting Information**

**S1 Fig.** *Cx. quinquefasciatus* and *Ae. albopictus* are permissive to YCV infection when fed a blood meal

**S2 Table.** Characterization of the running water and sewage

**S3 Fig.** YCV effects the growth of DENV-2, ZIKV and JEV in C6/36 and Aag2 cells during single- and coinfections under simultaneous infections

**S4 Fig.** YCV effects the growth of DENV-2, ZIKV and JEV in C6/36 cells during single- and coinfections under sequential infections
